# State-of-the-art review: The value of leveraging evidence and data (LEAD) in pediatric screening for familial hypercholesterolemia

**DOI:** 10.1016/j.ajpc.2025.101262

**Published:** 2025-08-22

**Authors:** Jonathan N Flyer, Tomáš Freiberger, Adam L Ware, Amy L Peterson

**Affiliations:** a Department of Pediatrics, The Robert Larner MD College of Medicine at the University of Vermont, Given Courtyard 2 South, 89 Beaumont Avenue, Burlington, VT 05405 USA; b Centre for Cardiovascular Surgery and Transplantation, Pekařská 53, Brno, 603 00, Czech Republic; c Faculty of Medicine, Masaryk University, Kamenice 5, 625 00 Brno, Czech Republic; d Division of Pediatric Cardiology, University of Utah, 81N. Mario Capecchi Drive, Salt Lake City, UT 84106 USA; e Department of Pediatrics, Division of Pediatric Cardiology, University of Wisconsin School of Medicine and Public Health, 600 Highland Ave, Madison, WI 53792 USA

**Keywords:** Pediatric lipid screening, Familial hypercholesterolemia, Guideline implementation, Improvement science

## Abstract

Familial hypercholesterolemia (FH) is a common genetic disorder of lipid metabolism resulting in lifelong elevated levels of low-density lipoprotein cholesterol and early atherosclerotic cardiovascular disease. Although FH can be identified at young ages, it remains frequently undetected, underdiagnosed, and undertreated in the United States and around the world. Despite compelling data to support screening for FH in children, and universal lipid screening guidelines endorsed by the National Heart, Lung and Blood Institute and the American Academy of Pediatrics, screening practices in the United States remain controversial and suboptimal. The Family Heart Foundation launched its LEAD (Leverage Evidence and Data) Pediatric Initiative at the 2024 Family Heart Foundation 10th Annual Global Summit to help understand testing barriers, propose innovative solutions, and integrate improvement science to measure outcomes. Presentations highlighted common challenges with pediatric lipid screening and demonstrated creative process solutions to improve screening prevalence for children. This state-of-the-art review discusses common barriers to pediatric lipid screening, identifies process solutions, and explores innovative practices to increase the frequency of universal pediatric lipid screening.

## 1 Introduction

Familial hypercholesterolemia (FH) is, in the heterozygous state, a common inherited genetic metabolic disorder of lipid metabolism, with a prevalence of approximately 1 in 300 individuals [1,2]. FH is characterized by lifelong elevated levels of low-density lipoprotein cholesterol (LDL-C) and is associated with increased risk of premature atherosclerotic cardiovascular disease (ASCVD) [3,4]. Individuals who have a pathogenic variant in the *LDLR, APOB* or *PCSK9* gene associated with FH carry a significantly greater risk of myocardial infarction compared to the general population, even when LDL-C levels are matched [5]. Although FH can be identified at young ages, it remains frequently undetected, underdiagnosed, and undertreated in the United States [6]. Untreated FH increases lifetime ASCVD risk through repeated, cumulative, and prolonged abnormal LDL-C exposure [7]. The Centers for Disease Control and Prevention identified FH as a Tier 1 genetic disorder with clear rationale and positive public health implications for screening and treatment [8,9].

**Fig. 1:**
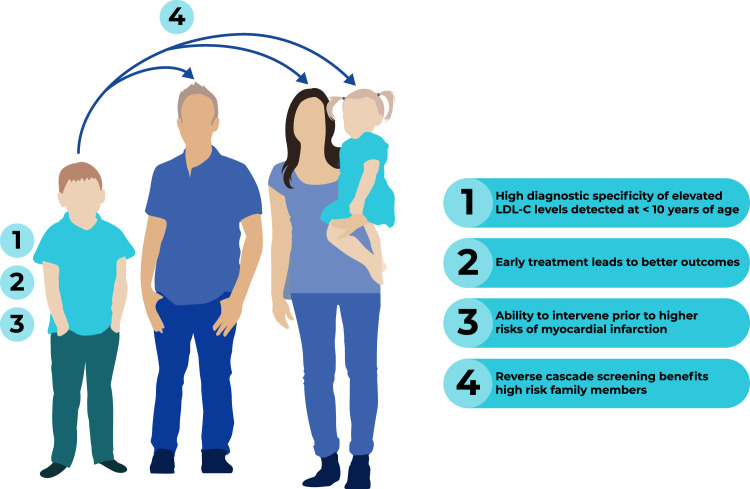
Rationale for familial hypercholesterolemia screening in childhood. Pediatric lipid screening is compelling, can identify children and family members at risk of atherosclerotic cardiovascular disease, and present options for earlier treatment. LDL-C: low density lipoprotein cholesterol.

The rationale for initiating FH screening *in childhood* is particularly compelling (Fig. 1). Currently, universal screening can be performed in children by measuring their LDL-C level. Confirmation of their FH diagnosis can be done with genetic testing, although this is not necessary to clinically diagnose or treat FH [5,10,11]. Significantly elevated LDL-C levels detected below the age of 10 years provide high diagnostic specificity for FH, as they are less influenced by lifestyle factors or comorbidities than in adults [12]. Although individuals with FH are at the highest relative risk of myocardial infarction between the ages of 25 and 39, approximately 60 % of heterozygous FH cases are diagnosed only after the age of 40, by which time the opportunities for effective primary prevention are often missed [13,14]. In contrast, children diagnosed and treated early, prior to the development of vascular damage, exhibit substantially better long-term outcomes compared to those who start treatment in adulthood [15]. Oral medications used to lower LDL-C levels (statin medications are the first-line agent) are available, low cost, and effective at lowering ASCVD risk and extending life [15].

In addition to these direct benefits to the child, diagnosing affected individuals during childhood provides the opportunity to reverse-cascade screen the child’s relatives to further identify siblings, parents, and communities at greater ASCVD risk [16]. Despite the availability of effective and affordable diagnostic strategies and lipid-lowering therapies, fewer than 10 % of adult patients and 5 % of children with FH have been diagnosed, leaving the vast majority of affected individuals undiagnosed and inadequately treated [13,17]. This delay in diagnosis and treatment creates entirely preventable cardiovascular morbidity and mortality, underscoring the need for systematic screening.

Several different screening strategies have been introduced internationally [18]. Opportunistic testing, where cholesterol levels are measured during unrelated healthcare visits, is widely practiced but lacks consistency and has limited reach [19]. Selective screening—targeting individuals based on family history of hypercholesterolemia or premature cardiovascular disease—has traditionally been a central strategy, but suffers from poor sensitivity and specificity [20]. Accurate and standardized collection of family history data is rarely achieved in clinical practice, and even when attempted, a substantial number of children with FH may be missed. In contrast, universal screening—targeting all individuals within a defined age group, such as all children at a certain age—has been shown to be a more effective approach for systematically identifying index cases [21]. Once these children are identified, cascade testing can be applied to their relatives, creating a powerful combined strategy.

To facilitate identification of index cases and address this delay in FH diagnosis, the National Heart, Lung and Blood Institute (NHLBI) published universal pediatric lipid screening guidelines in 2011 [22]. These guidelines were endorsed by the American Academy of Pediatrics (AAP) and recommend universal lipid screening during childhood as part of an integrated cardiovascular health promotion schedule: screen once during 9 to 11 years of age and, if normal, repeat the screen once between 17 and 21 years of age. However, despite these guidelines, universal lipid screening to identify FH in general pediatric primary care settings is infrequently accomplished, with several large studies reporting screening prevalence of <10% [[23], [24], [25], [26]]. In 2016 and again in 2023, the United States Preventive Services Task Force (USPSTF) reviewed the data surrounding universal pediatric lipid screening and gave it an “I” recommendation, indicating the evidence is insufficient to recommend for or against the practice, as data from randomized controlled trials linking childhood cholesterol levels to adult cardiovascular outcomes are limited [27].

There is less controversy surrounding universal pediatric cholesterol screening outside the US; in contrast, there is now broad consensus among international experts and professional societies that universal screening for FH in childhood is both necessary and achievable. This approach is strongly endorsed by the Prague Declaration, which emerged from a high-level policy meeting under the auspices of the Czech European Union presidency [28]. The Declaration explicitly advocates for the implementation of universal FH screening in children across Europe, highlighting the urgent need to overcome policy and logistical barriers. It serves as a blueprint for countries to follow, recommending that universal pediatric screening be implemented in all national systems, with local adaptations based on each country’s healthcare infrastructure, laboratory capacity, geographic and socioeconomic factors, resource availability, and population health needs. The optimal screening strategy involves a combination of biochemical testing (LDL-C measurement) and molecular genetic analysis. Biochemical testing provides a rapid and cost-effective first step, while genetic confirmation offers diagnostic precision and facilitates family-based cascade screening [28].

Despite compelling evidence linking childhood high LDL-C to premature ASCVD events in adulthood, in the US universal pediatric lipid screening recommendations still remain unnecessarily controversial (Table 1) [3,16,22,27,[29], [30], [31]]. To discuss challenges and innovations in pediatric lipid screening, clinical and research leaders were invited to participate in the 2024 Family Heart Foundation 10th Annual Global Summit. The Family Heart Foundation launched its LEAD (Leverage Evidence and Data) Pediatric Initiative at the Summit to help understand testing barriers, propose innovative solutions, and integrate improvement science to measure outcomes. The LEADing goals were to improve both provider understanding of the importance of FH screening and adherence to national screening guidelines to enhance early detection of FH. The purpose of this review is to discuss common barriers to pediatric lipid screening, highlight innovation, and explore process solutions to increase the frequency of universal pediatric lipid screening.

**Table 1:** Universal lipid screening recommendations by national professional society.

Professional Society	Year	Population	Recommendation
National Heart, Lung and Blood Institute (NHLBI): Expert Panel on Integrated Guidelines for Cardiovascular Health and Risk Reduction in Children and Adolescents	2011	Integrated Cardiovascular Health Schedule: • age 9–11 years • age 17–21 years	Grade B: Strongly Recommended to obtain ULS once in each age category, review results with patient, and manage abnormalities with lipid algorithms as needed. • Measurements may be either non fasting non-HDL or FLP.
American Academy of Pediatrics	Endorses NHLBI statement		
American Heart Association	2015	• age < 20 years • Ideal: age 6 to 12 years (LDL-C levels improve discrimination)	ULS for FH is feasible. • Cascade screening: should be performed to identify all affected family members of index cases.
National Lipid Association	2015	• age 9–11 years	All children, regardless of general health or risk factors. • Repeat lipid screening every 5 years if normal.
United States Preventive Services Task Force (USPSTF)	2016, 2023	• age <20 years : Asymptomatic children and adolescents	Grade I: Current evidence is insufficient to assess the balance of ULS benefits and harms.
American Academy of Family Physicians	Endorses USPSTF statement		

Recommendations for universal lipid screening based on professional society, patient age, and grade of evidence. FH: familial hypercholesterolemia; FLP: fasting lipid panel; HDL: high density lipoprotein; non-HDL = TC – HDL; TC: total cholesterol; ULS: universal lipid screening.

## 2 Challenges to pediatric lipid screening

**Fig. 2:**
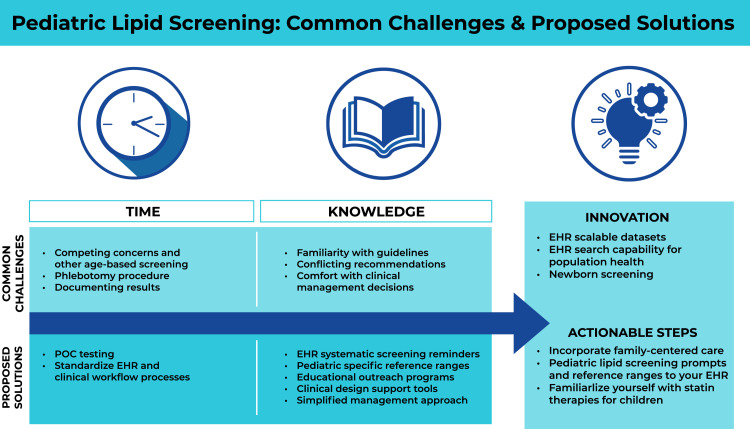
Central figure. Common challenges to pediatric lipid screening can be overcome with innovative process solutions.

Two main barriers to pediatric lipid screening in primary care have emerged over the past decade: 1) busy clinicians and families lack time needed for lipid screening; 2) limited understanding and comfort with pediatric lipid screening guidelines lowers its prioritization. This state-of-the-art review examines these barriers, discusses potential solutions within the primary care office, and introduces innovative systems approaches to improve lipid screening (Fig. 2).

### 2.1 Challenge: Busy clinicians and families lack time needed for lipid screening

Pediatric clinicians strive to provide comprehensive preventive screening as essential components of well-child care. However, the volume of necessary preventive screens remains a barrier to successfully increasing the frequency of pediatric lipid screening. Clinicians are tasked with triaging the extensive list of necessary and appropriate screens in addition to addressing family concerns that are raised during the visit. In most practices this all must occur in a period of 15 min of face-to-face time with the patient and family [32]. A study in adults evaluating the necessary time to complete all of the USPSTF recommended screenings suggested it would require 131 % of a physician’s working day [33]. While a similar study has not been completed in pediatrics, a comparable challenge is anticipated when reviewing the AAP recommended screenings for children between the ages of 9 and 11. This is especially true given tasks such as “Behavioral, Social, and Emotional Screening” and “Anticipatory Guidance” that often require careful discussion and time to address adequately.

Time challenges also extend to the pediatric patient and family. The need for phlebotomy to check a lipid panel has been previously established as a key barrier to pediatric lipid screening [[34], [35], [36]]. In the general pediatrics setting, a clinic-wide recommendation for routine lipid screening may falsely assume that families are prepared for venipuncture at the primary care well visit. In many primary care offices, phlebotomy includes the actual procedural time but often requires travel to a separate clinical location. It also may not account for additional time needed to perform venipuncture in children, who often have higher rates of avoidance behaviors due to needle phobia [37]. Whereas child life personnel and distraction elements are often helpful to mitigate needle phobia, few clinical settings have the additional time, resources, and expertise readily available to accommodate younger patients and overcome the challenge [38].

The time commitment does not end with the phlebotomy draw, however. Mild lipid abnormalities in children are common, and approximately 20 % of children will have an abnormal lipid level at the time of screening [39]. Clinicians and families may all feel overwhelmed with the need to address each abnormality with the pediatric patient and their family.

### 2.2 Potential solution: Point-of-care testing and standardized electronic health record documentation

Point-of-care (POC) testing for universal lipid screening is emerging as an efficient and effective alternative to overcome testing barriers posed by more traditional phlebotomy. In contrast to phlebotomy, POC diagnostics are performed by simple finger prick in the routine pediatric clinical setting and can be integrated within regular clinical workflows, hopefully leading to increased patient satisfaction [40]. POC procedures can be completed in a few minutes and by a variety of healthcare professionals, compared to longer laboratory processing wait times and the need for a specialized phlebotomist. Integrating POC lipid screening can increase health system efficiency as clinicians can provide patients and families with in-person results and consultation during routine care, reducing additional time and cost of follow-up care visits [41]. POC lipid screening offers an additional advantage of higher adherence; two recent studies have demonstrated up to 60 % testing completion rates utilizing POC compared to typically < 10 % with phlebotomy [42,43]. In contrast, a 5-year study of universal pediatric lipid screening in Wisconsin with in-building phlebotomy showed 69 % to 78 % of children for whom lipid screening was ordered completed the test [44,45].

Investigators from the University of Vermont developed quality improvement (QI) measures to increase the prevalence of lipid screening within an academic general pediatric cardiology practice over a 6-year period [42]. The project focused on universal lipid screening in youth 9–11 years and 17–21 years of age, independent of clinic visit reason or cardiovascular diagnosis. The primary SMART (Specific, Measurable, Achievable, Relevant, Timely) aim was to increase baseline screening prevalence from 7 % to >40 % within 4 years. The secondary aim was to implement POC testing processes and study their effect on adherence to NHLBI/AAP guidelines over an 18-month period.

A multidisciplinary pediatric cardiovascular QI team implemented a series of Plan-Do-Study-Act interventions over two phases at a single academic pediatric cardiology practice. In the first phase, the pediatric cardiologists: 1) measured regional interest from primary care teams about completing lipid screening in pediatric cardiology clinic; 2) offered phlebotomy testing at the conclusion of the cardiology visit; and 3) provided results and follow-up counseling separate from the initial visit. In the second phase, screening workflow processes were shifted to the beginning of the clinical visit to highlight the importance of lipid screening for cardiovascular health. The team standardized patient education materials, electronic health record (EHR) documentation, and physician counseling. Non-fasting POC testing was then incorporated and supervised by nursing during the clinical intake. Results were available for in-person review and discussion with the family. Clinical workflow evolved to standardize management of abnormal screens to align age-appropriate lab values, cardiovascular guidelines, and referral to a lipid clinic if needed.

There were 1467 patients with 1952 clinical visits during the 6-year study period. At project initiation, the baseline screening rate was 0 %, increasing to 7 % after the first phase. During the second phase, screening increased to 37 % with the change in the clinical workflows and then increased to 61 % with POC testing. Once available, POC testing became the predominant screening method compared to phlebotomy (91 % vs 8 %) and was used more commonly with 9–11-year-olds relative to 17–21-year-olds (88 % vs 70 %, *p* < 0.001). Over the entire study, the screening prevalence was similar between the two age groups (22 % of 9–11-year-olds vs 23 % of 17–21-year-olds, *p* = 0.62).

This study illustrates how improvement science can help modify clinical workflow to increase adherence to lipid screening guidelines and create sustainable results. In the past year, the methodology has been adapted to primary care practices and a quaternary pediatric cardiac center, who are now studying outcomes. This study also highlights the importance of partnership between pediatric cardiologists and primary care teams; 32 % of regional clinicians surveyed with this study provided project input, with nearly all (97 %) agreeing with pediatric cardiologists providing lipid screening and counseling. As the frequency of pediatric lipid screening remains suboptimal in all clinical settings, it is important to re-examine pediatric healthcare delivery to increase adherence, acceptability, and efficiency [[23], [24], [25], [26]].

### 2.3 Challenge: Limited understanding and comfort with pediatric lipid screening guidelines leads to lower prioritization of screening

Despite guidelines recommending universal pediatric cholesterol screening since 2011, many pediatric clinicians report limited understanding of screening guidelines, diagnosis of pediatric cholesterol abnormalities, and initiation of treatment [22]. In a large survey study of pediatricians, only 26 % reported feeling moderately or very knowledgeable about the 2011 NHLBI guidelines and 23 % felt lipid screening was a low priority [24].

In the survey of pediatricians, 55 % identified lack of local subspecialists for referral of dyslipidemia as a barrier to screening [24]. However, education among specialists is also still needed. In one survey study of pediatric cardiologists (subspecialty clinicians who are often called upon to care for children with dyslipidemia), 51 % reported little or no formal training on cholesterol disorders, 56 % underestimated the prevalence of FH by at least twofold, and only 54 % indicated they were at least “somewhat comfortable” prescribing statins to children [46].

### 2.4 Potential solution: Outreach and education regarding pediatric cholesterol screening with tools embedded in the electronic health record

Improving adherence to screening guidelines likely requires professional behavior changes, which are an active and continuous process. Examples of active interventions include educational outreach and systematic practice reminders, which are generally more favored compared to passive approaches [47]. Although QI interventions in cardiovascular medicine demonstrate promise for evidence-based testing and therapy endpoints, limited interventions have been applied to pediatric lipid screening [35,42,43,48,49].

In busy practice settings, a systematic approach to facilitate universal lipid screening is encouraged. Just as a member of the healthcare team is empowered to obtain anthropometric measurements and administer vaccines, an automated approach to checking a lipid panel can favorably increase screening frequency and identification of children at high-risk due to asymptomatic lipid disorders. Systems that facilitate the lipid panel order and follow with screening have the greatest impact on successful lipid screening [43]. In Wisconsin, clinician-focused educational sessions about pediatric cholesterol screening were offered followed by systematic modifications to the EHR, including adding age-appropriate reference ranges to lipid panel results, embedding the recommendation for pediatric cholesterol screening into the pediatric well visit note template, and streamlining order placement in the EHR. Over the 5-year period of this study, the rate at which clinicians placed an order in the EHR for a child eligible for universal cholesterol screening increased from 8 % to 50 % [44].

Pediatric clinicians with special interest in this topic, including subspecialists with an emphasis on lipid disorders, can have an important impact in the successful implementation of lipid screening through positive interactions with primary care practices. Given the number of preventive screens necessary, ongoing education regarding the importance and the purpose of lipid screening must be provided to the medical home. Education should emphasize both the importance of population screening and the appropriate actions for high-risk individuals.

Focusing on FH as a primary reason for lipid screening is beneficial in both universal lipid screening and caring for children with higher dyslipidemia risk. Comparing lipid screening and the incidence of FH to newborn screening and the incidence of conditions diagnosed on that test can be useful [50]. Pediatric clinicians are well-versed in screening for high-impact but uncommon diseases, and may be further motivated to perform lipid screening once recognizing that the prevalence of FH far exceeds many other conditions for which they regularly screen. Additionally, emphasizing the clear ASCVD risk associated with life-time levels of very high LDL-C can decrease overall disease severity if FH is identified and treated during childhood.

Education reinforcing normal and abnormal lipid parameters and how to distinguish patients who only require ongoing lifestyle modification from those at the highest risk for early ASCVD who require pharmacotherapy (Table 2) may decrease the hesitation to screen for lipid abnormalities. A screening algorithm outlined by the American Heart Association outlines specific lipid parameters that may indicate the need for additional treatment. An emphasis on appropriate next steps for an LDL-C level of ≥130 mg/dL for high-risk patients and ≥ 160 mg/dL for others may simplify the treatment algorithm for busy pediatric providers [30]. For patients with LDL-C abnormalities that do not reach these thresholds, additional clinician education regarding lifestyle interventions and timing of repeat lab draws (typically 3 or 6 months) can be helpful to prevent excessive repeat lab visits. Clarifying the distinction between high and low risk lipid abnormalities, and providing actionable measures that enable primary care teams to modify the lifetime risk of ASCVD in children with FH, may help change perceptions regarding the importance of universal lipid screening.

**Table 2:** Risk stratification by lipid parameters and medical conditions.

Lifetime ASCVD Risk	Condition	LDL-C Treatment Threshold*
Higher Risk	Homozygous FH, diabetes mellitus (types 1 and 2), coronary aneurysms, end stage renal disease, transplant vasculopathy, stem cell recipient	≥ 130 mg/dL
Moderate Risk	Heterozygous FH, Severe obesity, hypertension, coarctation, elevated LP(a), Pre-dialysis kidney disease, chest radiation	≥ 160 mg/dL
At Risk	Obesity, insulin resistance with comorbidities (dyslipidemia, MAFLD, PCOS), white coat hypertension, cardiomyopathies, pulmonary hypertension, chronic inflammatory conditions, chemotherapy,	≥ 160 mg/dL

Adapted from de. Ferranti et al. Cardiovascular Risk Reduction in High-Risk Pediatric Patients: A Scientific Statement from the American Heart Association. Volume. Circulation. 2019.

*Treatment threshold adjustments can be lowered in the presence of additional cardiovascular risk factors and discussion with lipid specialists.

ASCVD: atherosclerotic cardiovascular disease; FH: familial hypercholesterolemia, LDL-C: low density lipoprotein cholesterol; MAFLD: metabolic dysfunction-associated fatty liver disease; PCOS: polycystic ovarian syndrome.

Although teaching sessions and outreach efforts are important and may lead to transient increases in screening prevalence, durable collaboration and system design are likely needed for persistent change. Lipid experts can assist with system improvements that lead to sustainable changes for medical homes including the systematic inclusion of orders and POC screening at well-child visits. While decisions regarding resources for primary healthcare systems may be beyond control of the lipid specialists, sharing successful implementation stories in the literature and assisting with treatment algorithms can positively influence practice priorities. EHR-integrated clinical decision support tools can improve adherence to universal lipid screening guidelines. Several studies have demonstrated that automated alerts embedded within the EHR substantially increase lipid screening prevalence during pediatric well-child visits. Interventions that link reminders directly to order sets yield the greatest improvements in test completion. In a recent study, one tool increased screening prevalence by streamlining the provider workflow at the point of care [51]. Similarly, QI initiatives have documented increases in screening prevalence from <10 % to >50 % following implementation of EHR-based interventions [43,44]. As health systems seek to improve pediatric FH detection, leveraging the EHR represents a high-impact, cost-effective strategy. In lipid screening, as in all improvement efforts, it is imperative that the healthcare system make it easy to do the right thing.

### 2.5 Innovative systems approaches to cholesterol screening: using the EHR and newborn screening systems to identify FH

#### 2.5.1 Using the EHR

At a system level, the EHR offers a scalable platform for identifying children with FH through two primary mechanisms: retrospective case finding and proactive screening facilitation. Retrospective identification leverages EHR-derived phenotypic data, including LDL-C levels, family history, and clinical features often in combination with machine learning and natural language processing to apply an FH scoring system. Early attempts to identify FH patients in the EHR were limited by high false positive rates when relying solely on LDL-C thresholds (e.g., >190 mg/dL), which lack specificity, particularly in pediatric populations.

More recent approaches integrate unstructured data extracted via natural language processing (free text documentation of premature ASCVD, family history, or other diagnostic criteria) and machine learning classifiers, resulting in improved precision. The Screening Employees And Residents in the Community for Hypercholesterolemia (SEARCH) study and the Flag, Identify, Network, Delivery (FIND) FH initiative demonstrated the feasibility of this approach in adults, with the latter reporting a positive predictive value of 0.88 using EHR-based algorithms across diverse healthcare systems [52,53]. EHR-linked biobanks further enhance diagnostic accuracy by combining clinical data with genomic information. The electronic medical records and genomics (eMERGE) network and similar initiatives have demonstrated that integrating genotype and phenotype data improves case finding and allows for better stratification of cardiovascular risk [54]. Dikilitas et al. showed that return of results based on genomic findings linked to EHR records led to improved risk identification and clinical management for individuals with FH. Although primarily applied in adult populations to date, these methodologies are technically adaptable to pediatric datasets directly or through cascade screening and represent a promising avenue for early, systematic FH detection at scale.

#### 2.5.2 Newborn screening

There is growing evidence that FH can be identified at birth. Notably, LDL-C levels measured in newborns are significantly associated with elevated LDL-C concentrations at 12–14 months of age, indicating the potential of early screening to identify individuals at risk [55].

Screening strategies suitable for high-throughput newborn screening systems that combine biochemical and genetic methods are now available. These include measurement of LDL-C and apolipoprotein B-100 from dried blood spots, followed by genetic testing in those with the highest lipid levels [56,57]. This targeted approach has proven effective in identifying affected newborns with pathogenic or likely pathogenic variants for FH, with 1 in 667 newborns diagnosed with FH [58]. Furthermore, promising data were presented at the Global FH Summit 2024 from a pilot project utilizing LDL-C measurement from umbilical cord blood, followed by genetic testing in neonates with LDL-C levels above the 75th percentile. In a cohort of nearly 6000 newborns, this approach identified approximately one FH case per 400 infants screened at 12 different centers, demonstrating both feasibility and diagnostic yield (Freiberger T, Vrablik M, Majek, O. Pilot Project of Familial Hypercholesterolemia Screening in Newborns in the Czech Republic (CzeCH-IN). NCT05638022 - unpublished results).

Newborn FH screening offers several unique advantages, along with certain limitations. Among its key benefits is the early identification of homozygous FH—a rare but severe condition that requires aggressive lipid-lowering treatment in infancy to prevent the development of early and rapidly progressive atherosclerosis that can cause ASCVD events in childhood [59,60]. At the same time, it enables the detection of heterozygous FH, which is substantially more prevalent. While pharmacological treatment for heterozygous FH typically begins between 8 and 10 years of age, lifestyle measures introduced from as early as 2 years of age can provide long-term cardiovascular benefit [[61], [62], [63]]. Importantly, identifying FH in a newborn will lead to the detection of the condition in one of the parents, typically at an age when their cardiovascular risk is highest compared to the general population [14]. This reverse cascade testing effect allows for timely diagnosis and intervention in adults who may otherwise remain undiagnosed until after experiencing a major cardiac event. A delay of ten years in initiating treatment**,** for example if screening is conducted in 9- to 11-year-old children rather than at birth, may have critical implications for the affected parent**.**

In this context, newborn screening confers health benefits not only to the child, but also to the immediate family, aligning with the "family benefit" principle highlighted in public health policies for genetic disease screening. As emphasized by the European Organization for Rare Diseases, early detection of treatable conditions in parents is essential for ensuring the well-being of the entire family [64]. FH screening in newborns has the potential to trigger early diagnosis and preventive care in multiple affected relatives, thereby multiplying the impact of a single screening event. Nevertheless, while early results are promising, additional studies and real-world evidence are needed before newborn screening for FH can be endorsed as a fully validated public health strategy.

## 3 Everyday ways to LEAD the effort

International collaborations are encouraged for continued improvements in pediatric lipid screening prevalence and clinical outcomes. Networks of cardiovascular researchers, clinicians, and patient care advocates can enhance earlier FH detection, education, and treatment.

### 3.1 LEAD pediatric initiative

The 10th Annual Family Heart Global Summit was convened in September 2024 as a three day in-person conference to engage national experts in clinical care and research and share patient experiences with FH and elevated lipoprotein(a). The LEAD initiative was embedded into the Global Summit, and invited pediatricians, pediatric cardiologists, quality improvement and implementation science experts, and family advocates to a half-day launch session. Universal pediatric lipid screening barriers were discussed to promote process improvement and reinvigorate interest in national guideline adherence. The LEAD session acknowledged screening challenges from three distinct but related perspectives: patients and families, clinicians, and our healthcare system. Improvement science frameworks were provided to foster discussion and seek active solutions. Following the launch meeting, groups continued to collaborate virtually and develop QI initiatives in primary care, educational outreach, and advocacy. With the support of the Family Heart Foundation, the LEAD groups are working to continue to develop strategies to improve NHLBI/AAP guideline implementation.

### 3.2 Actionable steps to find FH in children

Early detection and treatment of FH is of paramount importance for both the child and family. Although universal lipid screening is predominantly intended for the primary care setting, all clinicians may actively encourage, endorse, and organize lipid screening for a child. It is important to identify practical and actionable starting points to help clinicians screen. A few introductory steps to start your practice include:

• In person screening: incorporate family-centered care and reduce testing barriers whenever possible.
• Information technology: make sure your EHR has appropriate pediatric reference ranges for lipid values and ideally, prompts to screen children with no prior lipid panel documented.
• Therapy: familiarize yourself with one or two statins and become comfortable prescribing them for children with FH.

## 4 Conclusion

FH is a common worldwide genetic lipid disorder that carries greatly increased ASCVD risk for children and their families. Early FH detection at young ages is of paramount importance to provide critical and directed medical therapy to reduce lifelong ASCVD and identify additional at-risk family members. Despite important recommendations to support pediatric lipid screening, common barriers continue to compromise effective testing in childhood, including the challenges of time needed for testing and the limitations of understanding the importance of screening at young ages. Such barriers may be addressed through shared process solutions: encouraging POC testing, standardizing EHR interactions, and initiating educational outreach programs. Innovative healthcare systems and processes to improve pediatric lipid screening prevalence can be further enhanced by ongoing collaborative clinical and research endeavors in support of patients and their families.

## CRediT authorship contribution statement

**Jonathan N Flyer:** Data curation, Investigation, Project administration, Resources, Software, Supervision, Visualization, Writing – original draft, Writing – review & editing. **Tomáš Freiberger:** Data curation, Investigation, Visualization, Writing – original draft, Writing – review & editing. **Adam L Ware:** Conceptualization, Visualization, Writing – original draft, Writing – review & editing. **Amy L Peterson:** Conceptualization, Project administration, Supervision, Visualization, Writing – original draft, Writing – review & editing.

## Declaration of competing interest

The authors declare the following financial interests/personal relationships which may be considered as potential competing interests:

Tomáš Freiberger MD PhD reports financial support was provided by Project National Institute for Research of Metabolic and Cardiovascular Diseases (Programme EXCELES. Outside the submitted work: Tomáš Freiberger MD PhD received honoraria and consultancy fees from Novartis, Sanofi, Sobi and Medison; Amy L Peterson MD MS received honoraria and consultancy feeds from Novartis, Mirum, and Regeneron. If there are other authors, they declare that they have no known competing financial interests or personal relationships that could have appeared to influence the work reported in this paper.
